# Resurgence of an Inborn Attraction for Animate Objects via Thyroid Hormone T_3_

**DOI:** 10.3389/fnbeh.2021.675994

**Published:** 2021-04-19

**Authors:** Elena Lorenzi, Bastien Samuel Lemaire, Elisabetta Versace, Toshiya Matsushima, Giorgio Vallortigara

**Affiliations:** ^1^Center for Mind/Brain Sciences, University of Trento, Rovereto, Italy; ^2^Department of Biological and Experimental Psychology, School of Biological and Chemical Sciences, Queen Mary University of London, London, United Kingdom; ^3^Department of Biology, Faculty of Science, Hokkaido University, Sapporo, Japan

**Keywords:** animacy, thyroid hormone, avian, sensitive period, plasticity, T_3_

## Abstract

For inexperienced brains, some stimuli are more attractive than others. Human neonates and newly hatched chicks preferentially orient towards face-like stimuli, biological motion, and objects changing speed. In chicks, this enhances exposure to social partners, and subsequent attachment trough filial imprinting. Early preferences are not steady. For instance, preference for stimuli changing speed fades away after 2 days in chicks. To understand the physiological mechanisms underlying these transient responses, we tested whether early preferences for objects changing speed can be promoted by thyroid hormone 3,5,3′-triiodothyronine (T_3_). This hormone determines the start of imprinting’s sensitive period. We found that the preference for objects changing speed can be re-established in female chicks treated with T_3_. Moreover, day-1 chicks treated with an inhibitor of endogenous T_3_ did not show any preference. These results suggest that the time windows of early predispositions and of sensitive period for imprinting are controlled by the same molecular mechanisms.

## Introduction

Early experience plays a crucial role in shaping neural and behavioural development. However, early experience effects are stronger during certain periods of development (Hensch, 2005). An example is provided by filial imprinting (Spalding, 1873; Hess, 1959; Bateson, 1979; Horn, 2004; McCabe, 2013; Vallortigara and Versace, 2018). Shortly after birth or hatching, the young of some animals, usually of precocial species, learn to recognise their social partners (e.g., the mother and siblings) by simply being exposed to them. In the young domestic fowl (*Gallus gallus*), for instance, imprinting usually occurs within 24–48 h from hatching. Lorenz (1935) used the term “critical period” to refer to the fact that rather than being available throughout the lifespan, filial imprinting is shown only during a limited period of life. This time window can be extended for a few more days when a proper stimulus is not immediately available (Bolhuis, 1991). Being a flexible period with the characteristics of a self-terminating process (Bolhuis, 1991), imprinting is now better known as a “sensitive period.” Sensitive periods are windows of plasticity during brain development, in which experience has a powerful effect (Hensch, 2005; Dehorter and Del Pino, 2020).

The thyroid hormone 3,5,3′-triiodothyronine (T_3_) is implicated in the timing of the sensitive period for imprinting (Yamaguchi et al., 2012). In domestic chicks, imprinting causes a rapid inflow of T_3_ in the brain, particularly in the intermediate medial mesopallium (IMM), an associative telencephalic region involved in learning the features of the imprinting object (Horn, 2004; Yamaguchi et al., 2012). Via Wnt-signalling pathway, the enzyme Dio_2_ (type 2 iodothyronine deiodinase), localised in vascular endothelial cells of the brain, converts the inactive form thyroxine (T_4_) into the active form T_3_ (Yamaguchi et al., 2012, 2018). Endogenous T_3_ level in the brain peaks around the peri-hatch period, and decays within a few days if not boosted by imprinting. Injection of iopanoic acid (IOP), a potent inhibitor of Dio_2_, impairs visual imprinting during the sensitive period (Yamaguchi et al., 2012). After the sensitive period, administration of exogenous T_3_ allows non-imprinted chicks to imprint, re-opening the sensitive period for memory formation (Yamaguchi et al., 2012).

Re-opening of sensitive periods has been obtained also by other pharmacological agents (Batista et al., 2016, 2018; Yamaguchi et al., 2016; Aoki et al., 2018), and it has been discovered for phenomena other than filial imprinting, such as ocular dominance in non-human mammals (Hensch and Quinlan, 2018) and absolute pitch in humans (Gervain et al., 2013).

Although imprinting can occur with either naturalistic stimuli (resembling a conspecific) or artificial objects, a large amount of evidence shows the existence of spontaneous unlearned preferences for animate features (Rosa-Salva et al., 2021). These preferences act as a sort of canalisation mechanism to direct the newborns’ attention, favouring exposure to stimuli that are more likely to be social partners (Di Giorgio et al., 2017; Versace et al., 2018; Rosa-Salva et al., 2021). Preferences for animacy cues, that set apart animate from non-animate objects, have been described in newly hatched chicks, comprising, e.g., preferences for face-like stimuli (Rosa-Salva et al., 2010), biological motion stimuli (Vallortigara et al., 2005; Miura and Matsushima, 2016; Miura et al., 2020) and self-propelled objects that move with variable speed (Rosa-Salva et al., 2016; for review see: Di Giorgio et al., 2017; Lorenzi and Vallortigara, 2021; Vallortigara, 2021). The same animacy cues operate on human newborns and other species, in particular for the preference for speed changes we are dealing with here (see in the human newborns Di Giorgio et al., 2021 and see for reviews: Di Giorgio et al., 2017; Lorenzi and Vallortigara, 2021; Rosa-Salva et al., 2021; Vallortigara, 2021).

These biological priors, whose main function seems to speed up learning by canalising imprinting, also operate only during transient windows of sensitivity in development. Visually naïve chicks show a spontaneous preference for the head (face-like) region of a stuffed hen during the first 2 days post-hatching, which then fades away on day 3 (Johnson et al., 1989). The spontaneous preference for objects moving with visible speed changes (Rosa-Salva et al., 2016) shows a window of sensitivity in three genetically selected and isolated breeds of chicks for only the first day of life, then disappearing on day 3 (Versace et al., 2019). Similarly, the biological motion preference occurs only within the first few days of life (Miura and Matsushima, 2012). Importantly, appearing slightly later on day 2 of life, biological motion preference exhibits also sex differences, with females being choosier than males when approaching a biological motion stimulus (Miura and Matsushima, 2012).

From the second day of life, precocious sexually dimorphic behaviours start to emerge in chicks (Andrew, 1966). Due to different levels of social motivation and aggression, males and females exhibit different attitudes towards familiar and unfamiliar individuals (Cailotto et al., 1989; Vallortigara et al., 1990; Vallortigara, 1992; Versace et al., 2017; Lemaire et al., 2020; Santolin et al., 2020). Progressively, females develop strong social cohesive behaviours with familiar subjects, while males engage more in aggressive and explorative ones (McBride and Foenander, 1962; McBride et al., 1969). At the neurobiological level, imprinting-related gene expression in the brain shows remarkable differences between sexes (Yamaguchi et al., 2012); among others, an upregulation of Dio_2_ gene enabling imprinting, which appears to be quantitatively different between males and females (Yamaguchi et al., 2012).

Here we report that the thyroid hormone T_3_ can modulate the timing of one window of early preferences for animacy cues, i.e., change of speed. Different from the sensitive period for imprinting, this preference does not depend on a particular experience with stimuli but rather canalises the animal’s attention towards particular stimuli, working as a spontaneous, unlearned biological prior.

We devised three different experimental conditions. The first was aimed to confirm that the same window of sensitivity for the spontaneous animacy preference conveyed by visible speed changes shown in genetically selected strains (Versace et al., 2019) also exists in the strain of broiler chicks we were using in the lab. We confirmed that the preference is there on post-hatching day 1, but fades away on day 2. To check whether T_3_ influences the duration of this window of sensitivity, we performed two experiments. First, in order to show that inhibition of endogenous T_3_ action can abolish the animacy preference, we injected the inhibitor IOP on post-hatching day 1, and compared IOP-injected chicks with vehicle-injected peers. Second, in order to show that the animacy preference can be re-established by exogenous T_3_ administration, we injected chicks with T_3_ on post-hatching day 3 and compared T_3_-injected chicks with vehicle-injected peers.

## Results

**FIGURE 1:**
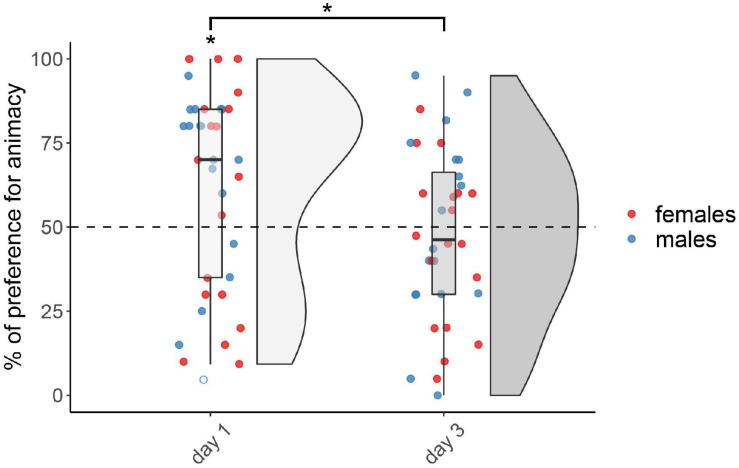
Preference for animacy for the not-injected chicks tested 1 or 3 days after hatching. To best represent the data, we used raincloud plots. Asterisks indicate significant differences from chance (black dotted line). Black asterisk between the two groups indicates a significant difference between testing Days. * indicates *p* < 0.05. Filled red dots represent female and blue male subjects, empty dots represent outliers, which were removed from the analyses.

The results for the not-injected condition are shown in Figure 1. The permutation test revealed a significant main effect of testing Day (*F*_(__1,67__)_ = 5.40, *p* < 0.05), but did not reveal any effect of Sex (*F*_(1,67)_ = 1.17, *p* = 0.28) nor interaction (*F*_(1,67)_ = 0.05, *p* = 0.83; see Supplementary Table 1 for the number of subjects tested in each condition for each sex, and for results split by sex). Chicks tested on day 1 showed a significant preference for animacy (*V*_(35)_ = 458, *p* < 0.05, *d* = 0.44), whereas chicks tested on day 3 did not show any preference (*V*_(36)_ = 310, *p* = 0.72, *d* = 0.08).

**FIGURE 2:**
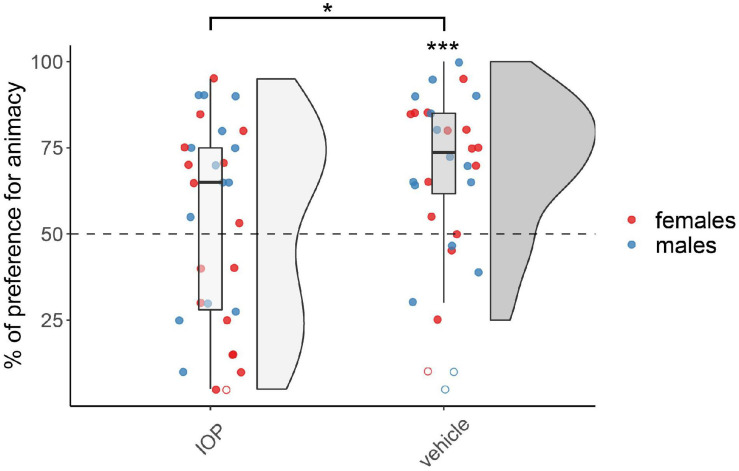
Preference for animacy for the chicks tested 1 day after hatching injected with IOP or with vehicle. To best represent the data, we used raincloud plots. Asterisks indicate significant differences from chance (black dotted line). Black asterisk between the two groups indicates a significant difference between IOP- and vehicle-injected chicks. *** indicates *p* < 0.001, * indicates *p* < 0.05. Filled red dots represent female and blue male subjects, empty dots represent outliers, which were removed from the analyses.

The results for chicks treated on day 1 with the IOP (Dio_2_-inhibitor) are shown in Figure 2. The permutation test revealed a significant main effect of Treatment (*F*_(1,54)_ = 5.74, *p* < 0.05), but did not reveal any effect of Sex (*F*_(1,54)_ = 1.10, *p* = 0.30) nor interaction (*F*_(1,54)_ = 0.66, *p* = 0.42; see Supplementary Table 1). The vehicle-injected group showed a significant preference for animacy (*V*_(28)_ = 347, *p* < 0.001, *d* = 1.01), whereas the IOP-injected group did not show any preference (*V*_(30)_ = 268.5, *p* = 0.46, *d* = 0.14). As expected, at this age no sex differences were apparent.

**FIGURE 3:**
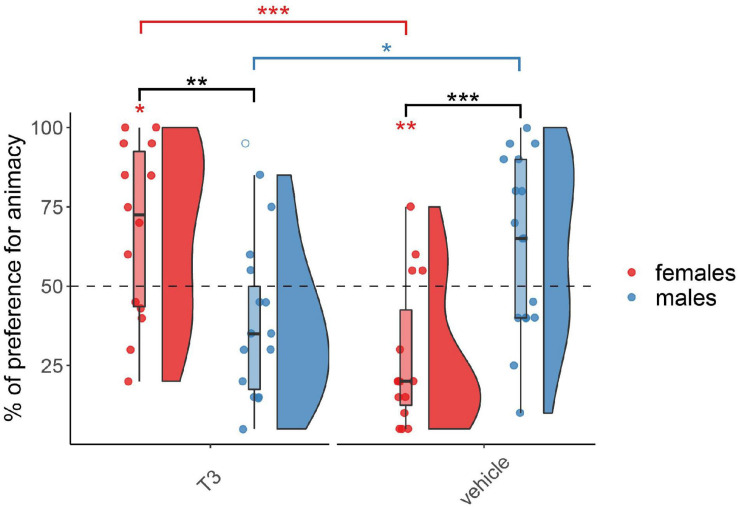
Preference for animacy for the chicks tested 3 days after hatching injected with T_3_ or vehicle. To best represent the data, we used raincloud plots. Black asterisks indicate a significant difference between sexes (females red and males blue) within each injection group. Red asterisks above a red horizontal line indicate a significant difference between females of each injection group. Blue asterisks above a blue horizontal line indicate a significant difference between males of each injection group. Red asterisks indicate a significant difference from chance in females. *** indicates *p* < 0.001, ** indicates *p* < 0.01, * indicates *p* < 0.05. Filled red dots represent female and blue male subjects, empty dots represent outliers, which were removed from the analyses.

The results for the chicks injected with T_3_ on day 3 are shown in Figure 3. As expected, the permutation test revealed at this age a significant interaction between Treatment and Sex (*F*_(1,57)_ = 25.02, *p* < 0.001) but did not reveal any main effect of Treatment (*F*_(1,57)_ = 1.27, *p* = 0.26) or Sex (*F*_(1,57)_ = 0.20, *p* = 0.65). Females and males showed a different pattern within each group, T_3_-injected (*W*_(29)_ = 166.5, *p* < 0.01, *d* = 1.17) and vehicle-injected (*W*_(32)_ = 39.5, *p* < 0.001, *d* = 1.40). T_3_- and vehicle-injected females showed a significant difference in their preferences (*W*_(29)_ = 183.5, *p* < 0.001, *d* = 1.59). T_3_-injected females showed a significant preference for animacy (*V*_(14)_ = 85, *p* < 0.05, *d* = 0.63), whereas vehicle-injected females showed a significant preference for the non-animacy stimulus (*V*_(15)_ = 11, *p* < 0.01, *d* = 1.0). T_3_- and vehicle-injected males also showed a marginally significant difference in their preferences (*W*_(32)_ = 61, *p* < 0.05, *d* = 0.98). However, in spite of a trend for an inverted pattern with respect to females, T_3_-injected males did not show any significant preference for the stimuli (*V*_(15)_ = 27, *p* > 0.05, *d* = 0.53) nor did vehicle-injected males (*V*_(17)_ = 116, *p* > 0.05, *d* = 0.47).

## Discussion

Untreated chicks showed a clear spontaneous preference for visible speed changes, a self-propelled cue to animacy (Rosa-Salva et al., 2016), on post-hatching day 1 that disappeared on day 3. Restricted sensitivity windows to animacy motion cues seem thus to exist in chicks similar to those for the head region of the mother hen and for biological motion (Johnson et al., 1989; Miura and Matsushima, 2012). Similar sensitivity windows have been described in humans as well. During the first month of life infants preferentially look at schematic face-like configurations over identical stimuli in a non-face configuration, whereas the same is not observed at 3 and 5 months of age (Johnson et al., 1991). Also, the intensity of selective EEG responses to face-like patterns tends to decrease over time after the first hours of life (Buiatti et al., 2019).

The thyroid hormone T_3_ appears to play a crucial role in the sensitivity window for spontaneous motion animacy preference. Animacy preference disappeared on day 1 in chicks injected with IOP, an inhibitor of Dio_2_ the enzyme converting the inactive form T_4_ into the active T_3_. While vehicle-injected day 1 chicks had a significant preference for animacy, consistently with previous findings (Rosa-Salva et al., 2016).

On day 3, administration of T_3_ seems to restore animacy preference, at least in females. Sex differences, which are well-known to be associated with critical period regulation by thyroid hormones (see Miura and Matsushima, 2012; Yamaguchi et al., 2012; Batista and Hensch, 2019), seem to be complicated at this age also by the effect of the injecting needle in itself. T_3_-injected females preferred the animacy stimulus, while vehicle-injected females preferred the non-animacy one. Given that we do not expect any physiological effect of vehicle (as indeed this is the case for day-1 treated chicks), it seems that in older chicks the mere, though mild, pain experience of injecting the needle interacts with the expression of animacy preferences at least in females (males showed a somewhat similar effect but with inverted direction).

Precocious sex differences in social motivation and aggression are commonly observed in chicks (Andrew, 1966). Functionally, it has been argued that they may arise from different levels of social motivation (Cailotto et al., 1989; Vallortigara et al., 1990) and attitudes towards novelty (Vallortigara, 1992) in the two sexes. Females tend to engage more than males in social reinstatement and usually prefer to approach familiar individuals (Cailotto et al., 1989; Vallortigara et al., 1990; Vallortigara, 1992), whereas males tend to approach unfamiliar ones (Vallortigara, 1992; Versace et al., 2017; Santolin et al., 2020). In natural populations fowls exhibit territorial behaviour wherein single dominant cocks maintain and patrol a large territory within which several highly social-aggregated females live (McBride and Foenander, 1962; McBride et al., 1969). This social organisation may favour the prevalence of gregarious and affiliative behaviours in females and aggressive and exploratory behaviours in males.

Sex differences have also been described for biological motion preferences, whose window of sensitivity occurs exactly around day 3 (Miura and Matsushima, 2012). Therefore, it could be that the injection of T_3_ affected both sexes similarly, but that they exhibited different behavioural responses coherent with their natural preferences that become apparent from day 3. Females would thus preferentially approach the animacy stimulus, engaging in social reinstatement with the most familiar, as predisposed, object. Whereas males, less socially motivated, would engage in explorative responses towards the unfamiliar, non-predisposed, object.

It could also be that the efficiency of T_3_ transport from muscle through blood vessels and finally to the brain is different between males and females. This may point towards a possible limitation of the method adopted here and suggests different T_3_ administration strategies.

From a neurobiological perspective, sex differences related to imprinting have been found in the brain. Several genes upregulate during imprinting, but only some do it in a sex dimorphic way. The Dio_2_ gene expresses more in males than in females during imprinting, while Tubby-like protein 1 gene, producing an upstream cell signalling protein, expresses more in females than males (Yamaguchi et al., 2012). Such differences might underly the behavioural differences between the two sexes observed in the present study in older chicks.

Note that vehicle-injected females on day 3 preferred the non-animacy stimulus, whereas males had a trend to prefer the animacy one. From day 3, chicks start to be more fearful due to the rising of hormonal levels (Schaller and Emlen, 1962; Rogers, 1995). Avoidance behaviours increase progressively from day 3 on, but with different timings in the two sexes. Males show a weaker avoidance response than females until post-hatching day 4 (Schaller and Emlen, 1962). Sex differences observed in the vehicle group on day 3 might arise from the different reactions to the needle’s stressful event. The fear caused by the injection in females might have evoked an avoidance response towards the most animate object, the animacy stimulus, making them walk to the opposite end of the apparatus. In males, less avoidant and more prone to aggression at this age, the same event could have evoked a tendency to approach the most animate stimulus.

It is also worth noting that early during development the levels of different thyroid hormones (T_3_ and T_4_, thyroxine) in the brain show sex-dependent differences. The onset of the surge of T_4_ in male zebra finches (*Taeniopygia guttata*) precedes that of females, while the onset of T_3_ in females precedes that of males (Yamaguchi et al., 2017). Strengthening the hypothesis of an interaction between T_3_ and other sexually dimorphic hormones, response to animacy in visually naïve chicks involves brain regions rich in sex steroid hormone receptors (Mayer et al., 2016, 2017a,2017b; Lorenzi et al., 2017), part of the so-called *Social Behavior Network*, which appears to be highly conserved in vertebrates (Newman, 1999; O’Connell and Hofmann, 2011; Goodson and Kingsbury, 2013; Lorenzi et al., 2017).

Thyroid hormones are key regulators of vertebrates’ brain development (Van Herck et al., 2013). Among others, T_3_ directly influences the expression of genes by binding to its nuclear receptors, which act as transcription factors (Harvey and Williams, 2002). Therefore, abnormalities in the level of T_3_ during development may result in permanent impairments. In the present study, inhibiting T_3_ function gave rise to a lack of the spontaneous approach behaviour towards animacy stimuli. Abnormalities in spontaneous preferences for animacy at birth have been linked to autistic spectrum disorder (Sgadò et al., 2018; Lorenzi et al., 2019). Interestingly, human neonates at high familial risk for autism exhibit anomalous preferential looking patterns to animacy cues provided by schematic face-like and biological motion stimuli (Di Giorgio et al., 2016).

## Conclusion

In conclusion, we showed that providing exogenous T_3_ restores the predisposition to orient towards motion-animacy cue on day 3 post-hatch, whereas inhibiting the endogenous T_3_ conversion prevents the predisposition to appear on day 1. The similarity of thyroid hormone effects on imprinting and on early predispositions is unlikely to be coincidental. Furthermore, re-opening the neural plasticity to the effects of environmental stimuli without restoring at the same time the biological priors for proper environmental stimulation could appear worthless. The two processes should be functionally and temporally linked, and probably for this reason they share the same molecular ground.

## Materials and Methods

### Animals

All experiments were carried out in compliance with the applicable European Union and Italian laws, and guidelines for animals’ care and use. All the experimental procedures were approved by the Ethical Committee of the University of Trento OPBA and by the Italian Health Ministry (permit number 1139/2015).

Fertilised eggs of the Aviagen Ross 308 strain were provided by a commercial hatchery (Azienda Agricola Crescenti, Brescia, Italy). Upon arrival, eggs were incubated under controlled temperature (37.7°C until post-hatching day 1, 33°C until post-hatching day 3) and humidity (60%) within incubators (FIEM MG140/200 Rural LCD EVO). Incubators were kept in darkness, preventing the chicks from any visual experience during incubation and hatching prior to testing. We employed a total of 190 domestic chicks (*G. gallus*; 95 females; the exact number of chicks in each condition and sex is shown in Supplementary Table 1). Sex was determined by feather dimorphism.

### Apparatus and Stimuli

**FIGURE 4:**
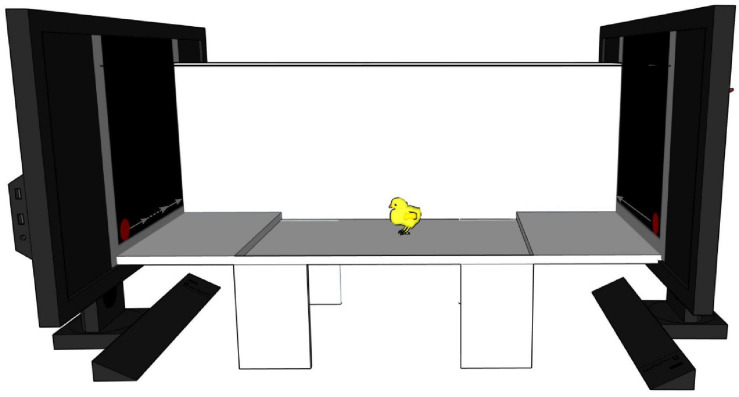
Schematic representation of the experimental apparatus. A white corridor with two video-screens at the two opposite ends playing the stimuli. On the left monitor is depicted the stimulus changing speed (dotted line represents faster speed), on the right monitor is represented the constant moving stimulus. The chick is represented in the starting area at the centre. Two little steps divide it from the two choice areas. For representative purposes one of the two longitudinal walls is represented translucent.

We used the same apparatus and stimuli as in previous studies investigating motion-animacy preference in newly hatched chicks (Rosa-Salva et al., 2016, 2018; Lorenzi et al., 2017, 2019). A detailed description of the apparatus, stimuli and procedure used can be found in Rosa-Salva et al. (2016) (Exp. 2). Briefly, the apparatus consisted of a white corridor (85 × 30 × 30 cm, see Figure 4) with two opposite high-frequency monitor screens (ASUS MG248Q, 24″, 120 Hz). Three areas subdivided the corridor: a central one (starting area: 45 cm long) and two lateral ones (choice areas: 20 cm long each). A small step (1.5 cm high) on each side delimited the boundaries between starting and choice areas. A video-camera, centrally located above the apparatus, recorded animals’ behaviour. The two stimuli were displayed on the screens and represented a red circle (diameter 3 cm) moving horizontally. One stimulus was moving at a constant speed (≈4.64 cm/s on our monitors) while the other one was visibly changing speed (the slower speed being ≈3.37 cm/s and the faster one being ≈19.64 cm/s), a reliable cue to animacy (Rosa-Salva et al., 2016). We counterbalanced the position of the stimuli on the screens between subjects.

### Intramuscular Injections

Iopanoic acid (IOP 10 mM, TCI I0300, Tokyo Chemical Industry Co., Ltd., Tokyo, Japan) was dissolved in 0.05M NaOH solution at 1 mM and rebuffered to pH = 8.5 by 6M HCl. 3,3′,5-Triiodo-L-thyronine (T_3_, 100 μM, Sigma-Aldrich, T-2877) was dissolved in 0.002M NaOH and 0.9% NaCl. Vehicle solutions were also prepared to control for any effect of the injections. Respectively, the vehicle for IOP was a 0.05M NaOH solution buffered to pH = 8.5 by 6M HCl, while the vehicle for T_3_ was a 0.9% NaCl and 0.002M NaOH solution (vehicle-injected groups). One hour before testing, each subject was carefully taken from the incubator in complete darkness. A black hood on the head prevented any source of visual stimulation during the intramuscular injection to thigh. Control chicks underwent the same procedure without receiving any injection (not-injected groups). Immediately after, each chick was placed back to the dark incubator. To distinguish single individuals in the darkness while keeping the same auditory environment experienced before injection, we placed the chicks in individual compartments within the same incubator.

### Testing

One hour after injection (according to the different groups assigned), each chick was individually tested for the spontaneous preference for animacy for 10 min. After placing each subject in the starting area, the time spent in each sector of the corridor was recorded. In order to measure the animal preference for motion-animacy, we considered the ratio (%) of time spent near the animacy stimulus over the total choice time using the formula:

$$\mathrm{Preference}\,\mathrm{for}\,\mathrm{animacy}=\frac{\mathrm{time}\,\mathrm{spent}\,\mathrm{close}\,\mathrm{to}\,\mathrm{animacy}}{\mathrm{time}\,\mathrm{spent}\,\mathrm{close}\,\mathrm{to}\,\mathrm{both}\,\mathrm{stimuli}}\times 100.$$

The preference score could range from 0% (full preference for the non-animacy stimulus) to 100% (full preference for animacy), while 50% represented the absence of preference. Permanence in the starting area for the total duration of the test was considered as no choice and led to the exclusion from further analyses (this occurred in about 47% of the cases). Sexing of the subjects occurred at the end of the procedure of testing.

### Statistical Analysis

The statistical analyses were performed using RStudio v1.1 and the following packages: *goftest* (Faraway et al., 2019), *nlme* (Pinheiro et al., 2020), *tidyr* (Wickham et al., 2020), *plyr* (Wickham, 2011), *dplyr* (Wickham et al., 2020), *reshape* (Wickham, 2007), *lsr* (Navarro, 2013), *ggplot2* (Wickham, 2016), and *lmPerm* (Wheeler and Torchiano, 2016).

The number of subjects required in each group was *a priori* determined with a power analysis (Champely et al., 2020) with an effect size of 0.96, and an alpha of 0.05. Results showed that 18 individuals were required per group and per sex to achieve a power of 0.80.

To detect the presence of outliers, we used a Multivariate Model Approach using Cook’s distance. Subjects having a Cook’s distance four times greater than the group mean were considered as outliers and discarded from further analyses (Kannan and Manoj, 2015). We identified six outliers from different groups. To assess the normality of data distribution, we looked at the distribution of residuals (Q–Q plot).

As parametric assumptions were not met, we used non-parametric tests. In not-injected condition, to determine whether the testing Day (two levels: day 1 and day 3) and Sex (two levels: female and male) affected animacy preference, we performed a permutation test using *F*-test probabilities. A similar test was conducted for the IOP- and T_3_-injected conditions, to determine whether the treatments (two levels: IOP/T_3_ and respective vehicles) and Sex (Two levels: female and male) affected animacy preference.

To determine whether the preference was statistically different between groups within each condition (not-, IOP-, T_3_-injected), we conducted two-sample Wilcoxon tests. To examine whether each group had a significant preference for either stimulus, we conducted one-sample Wilcoxon tests against chance level (50%). We calculated Cohen’s *d* (*d*) for each Wilcoxon test performed.

## Data Availability Statement

The raw data supporting the conclusions of this article will be made available by the authors, without undue reservation.

## Ethics Statement

The animal study was reviewed and approved by the Ethical Committee of the University of Trento OPBA and by the Italian Health Ministry.

## Author Contributions

EL, BL, TM, GV, and EV: conceptualisation. EL, BL, TM, and GV: methodology. BL: software. EL and BL: formal analysis, investigation, writing–original draft, and visualization. GV and TM: resources. GV, TM, and EV: writing-review and editing. GV: funding acquisition. All authors contributed to the article and approved the submitted version.

## Conflict of Interest

The authors declare that the research was conducted in the absence of any commercial or financial relationships that could be construed as a potential conflict of interest.
