# Supplementary material for: Resurgence of an Inborn Attraction for Animate Objects via Thyroid Hormone T3
Source: Front Behav Neurosci. 2021 Apr 19;15:675994. doi: 10.3389/fnbeh.2021.675994 (PMC8092046; doi:10.3389/fnbeh.2021.675994)
Supplement: Supplementary file 1 [file Table_1.DOCX]

| **Not-injected** | | **DAY 1** | | | | **DAY 3** | | | |
| --- | --- | --- | --- | --- | --- | --- | --- | --- | --- |
|  | | N | Mean | SD | SEM | N | Mean | SD | SE |
| Females | | 18 | 58.76 | 33.44 | 7.88 | 18 | 45.08 | 23.61 | 5.56 |
| Males | | 17 | 67.20 | 23.45 | 5.69 | 18 | 50.71 | 27.45 | 6.47 |
|  | |  |  |  |  |  |  |  |  |
| **IOP-injected** | | **IOP** | | | | **Vehicle** | | | |
| **DAY 1** |  | N | Mean | SD | SEM | N | Mean | SD | SE |
|  | Females | 16 | 48.36 | 29.55 | 7.39 | 14 | 69.40 | 19.29 | 5.15 |
|  | Males | 14 | 60.52 | 26.91 | 7.19 | 14 | 70.83 | 21.21 | 5.67 |
|  | |  | | | |  | | | |
| **T_3_-injected** | | **T_3_** | | | | **Vehicle** | | | |
| **DAY 3** |  | N | Mean | SD | SEM | N | Mean | SD | SE |
|  | Females | 14 | 67.36 | 27.57 | 7.37 | 15 | 27.33 | 22.67 | 5.85 |
|  | Males | 15 | 37.65 | 23.31 | 6.02 | 17 | 62.94 | 27.62 | 6.70 |

Table 1: Summary of subjects tested and animacy preference in each condition. Number of subjects tested in each condition (N), subdivided by sex, mean preference for animacy, standard deviation (SD) and standard error (SE).
